# Protein Solubility and Folding Enhancement by Interaction with RNA

**DOI:** 10.1371/journal.pone.0002677

**Published:** 2008-07-16

**Authors:** Seong Il Choi, Kyoung Sim Han, Chul Woo Kim, Ki-Sun Ryu, Byung Hee Kim, Kyun-Hwan Kim, Seo-Il Kim, Tae Hyun Kang, Hang-Cheol Shin, Keo-Heun Lim, Hyo Kyung Kim, Jeong-Min Hyun, Baik L. Seong

**Affiliations:** 1 Institute of Life Science and Biotechnology, Yonsei University, Seodaemun-Gu, Seoul, Korea; 2 Department of Biotechnology, College of Engineering, Yonsei University, Seoul, Korea; 3 Department of Pharmacology, School of Medicine, and Center for Diagnostic Medicine, Institute of Biomedical Science and Technology, Konkuk University, Seoul, Korea; 4 Department of Bioinformatics and Life Science, and CAMDRC, Soongsil University, Seoul, Korea; Baylor College of Medicine, United States of America

## Abstract

While basic mechanisms of several major molecular chaperones are well understood, this machinery has been known to be involved in folding of only limited number of proteins inside the cells. Here, we report a chaperone type of protein folding facilitated by interaction with RNA. When an RNA-binding module is placed at the N-terminus of aggregation-prone target proteins, this module, upon binding with RNA, further promotes the solubility of passenger proteins, potentially leading to enhancement of proper protein folding. Studies on *in vitro* refolding in the presence of RNA, coexpression of RNA molecules *in vivo* and the mutants with impaired RNA binding ability suggests that RNA can exert chaperoning effect on their bound proteins. The results suggest that RNA binding could affect the overall kinetic network of protein folding pathway in favor of productive folding over off-pathway aggregation. In addition, the RNA binding-mediated solubility enhancement is extremely robust for increasing soluble yield of passenger proteins and could be usefully implemented for high-throughput protein expression for functional and structural genomic research initiatives. The RNA-mediated chaperone type presented here would give new insights into *de novo* folding *in vivo*.

## Introduction

Folding of substantial fraction of newly synthesized proteins has been known to be assisted by molecular chaperones in the highly crowded cytosolic environment [1], [2]. Surprisingly, however, biochemical and genetic analyses have shown that only a limited number of proteins are dependent on the molecular chaperones [2]–[5], suggesting that other chaperone types and mechanisms might exist *in vivo*. Consistent with the restricted role of molecular chaperones, coexpression of molecular chaperones for the production of functional heterologous proteins in *E. coli* cytosol has been found effective only for limited cases [6]. Alternatively, fusion to highly soluble carriers such as maltose-binding protein (MBP) and NusA provides practical means to circumvent inclusion body formation [7]–[10]. Nevertheless, production of properly folded proteins of heterologous origin in *E. coli* host is still difficult, necessitating identification of more efficient folding vehicle for the high-throughput supply of functional proteins.

Molecular chaperones transiently bind to and shield the exposed hydrophobic surfaces by direct hydrophobic interactions and/or encapsulation to prevent misfolding and aggregation, leading to proper folding [2], [11]. On the other hand, charge is one of the crucial factors determining the solubility of proteins in the aqueous environment [12]–[14]. Electrostatic repulsions by charged residues can counteract intermolecular hydrophobic interactions of their linked residues [15]. Anionic tags promote solubility of their linked proteins [16], [17]. Consistently, the charges of fusion partners are closely correlated with their solubilizing ability [7], [18], [19]. These findings indicate that the hydrophobic shielding is not a sole determinant of stabilizing aggregation-prone folding intermediates against aggregation, and other mechanism may exist for folding of nascent proteins inside the cells.

Polyanions, including RNA and DNA, can accelerate the refolding rate of the Arc repressor dimer by nonspecific electrostatic interactions *in vitro* [20]. The intrinsically disordered proteins and domains form ordered structure or fold upon binding to its cognate RNA [13], [21]. In particular, ribosome and its component 23S rRNA have been reported to behave like molecular chaperones *in vitro* in a *trans*-acting mode [22], [23]. However, their relevance to *de novo* folding *in vivo* still remains largely unknown. All newly synthesized polypeptides are tightly linked to ribosomes during their biogenesis and folding process. Nevertheless, the roles of ribosomes in the aggregation and folding behavior of their linked aggregation-prone polypeptides in a *cis*-acting manner have been poorly understood. Notably, ribosomes are RNP complexes in which RNAs are major components and basic structural frames [24]. Thus, studies on the role of RNAs in the aggregation and folding behavior of their interacting proteins both *in vitro* and *in vivo* are required to understand *de novo* folding inside the cells.

**Figure 1:**
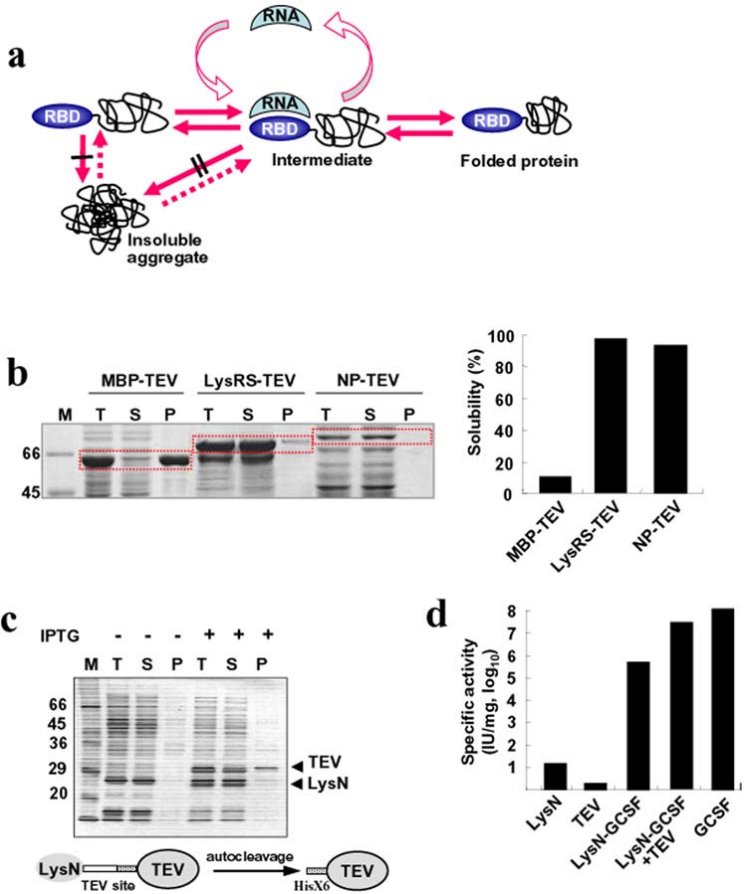
Development of RBPs as solubility enhancers. (a) Proposed model for RNA binding-mediated protein folding. Both the folded RBD at N-terminal position and bound RNA prevent inter-molecular interactions among folding intermediates, leading to soluble expression and favoring kinetic network into productive folding. The number of black bars (| and ∥) represents the extent of aggregation inhibition. (b) The comparison of solubility-enhancing ability by RBP with that of MBP. *E. coli* lysyl tRNA synthetase (LysRS) and influenza virus nucleoprotein (NP) were used as RBP to monitor the soluble expression of tobacco etch virus (TEV) protease. The solubility-enhancing ability of RBP was compared to that of MBP. The fusion proteins were expressed at 37°C and their solubility was analyzed by SDS-PAGE. M, T, S, and P represent molecular weight marker, total lysates, soluble fraction, and insoluble fraction, respectively. (c) Autocatalytic cleavage of LysN-TEV containing TEV cleavage sequence between LysN and TEV protease in *E. coli* cytosol. Non-induced (−) and IPTG induced (+) cell extracts were analyzed by SDS-PAGE. The uncleaved LysN-TEV was not detected clearly on SDS-PAGE due to efficient cleavage. (d) Cell proliferation assay of GCSF expressed as LysN-GCSF. The purified TEV protease described in Figure 1c was used to cleave the purified LysN-GCSF. The purified LysN, TEV protease, and LysN-GCSF before and after cleavage with TEV protease were compared with the GCSF standard in the cell proliferation assay as described in Methods.

Based on the apparent charge effect on protein solubility and the folding induced by RNA binding, here we provide evidence of RNA-interaction mediated protein solubility and folding enhancement. When an RNA-binding domain (RBD) is fused to target proteins, this domain, through binding with RNA, further promotes the solubility of downstream passenger proteins *in vivo*, potentially leading to a proper folding. The binding of highly negative-charged RNA to RBD-harboring proteins during folding process would promote the solubility and folding of whole proteins probably by virtue of the electrostatic repulsions caused by the bound RNA (Fig. 1a). In effect, RNA could exert efficient chaperoning effects on its bound proteins. In addition, RNA-binding protein (RBP) could be powerful solubility enhancer for high-throughput soluble expression of heterologous proteins through its interaction with RNA molecule.

## Results

### Development of RBPs as solubility enhancers

To explore potential chaperoning role of RNA for RBD-harboring proteins, we initially tested several RBPs, including *E. coli* lysyl tRNA synthetase (LysRS) [25], influenza A virus (WSN/3/33) nucleoprotein (NP) that exhibits non-specific RNA-binding properties [26], Ffh of signal recognition particle [27], C5 of RNase P [28], and Hsp 15 [29]. MBP, known as one of the best avenues to the soluble expression of fusion proteins so far [8], was included as control. As a reporter protein, tobacco etch virus (TEV) protease, mainly expressed as inclusion bodies without fusion in *E. coli* [8], was used.

LysRS and NP-fused TEV protease were predominantly expressed as a soluble form (≥90%) at 37°C, whereas MBP-fused TEV protease was marginally soluble, indicating that both LysRS and NP are much superior to MBP for promoting the solubility of TEV protease (Fig. 1b). The low expression of NP-TEV protease is due to the low expression of NP protein itself (data not shown) perhaps due to codon bias in *E. coli* host for the influenza virus derived protein. Hsp15-TEV protease was expressed as a soluble form (40%) at 37°C, and the solubility was greatly increased at 27°C (≥90%) (Fig. S1). Likewise, the solubility of C5-fused TEV protease was significantly increased at 27°C. All TEV fusion proteins exhibited site-specific protease activity as confirmed by the cleavage of LysN-fused human granulocyte colony-stimulating factor (LysN-GCSF) containing TEV cleavage site at the linker region (Fig. S2).

LysRS is a homodimeric protein (114 kDa), and its monomer consists of N-terminal (LysN) and C-terminal catalytic domains [30]. The LysN domain binds to the anticodon of tRNA^Lys^ [31] and was expected to serve as an independent RBD. We therefore investigated whether LysN as a single RBD (N-terminal 154 residues of LysRS) exhibits chaperoning activity toward its target proteins such as TEV protease and GCSF. From the initial LysN-TEV fusion construct separated by a linker sequence containing the TEV recognition site and histidine tag, two cleavage products corresponding to mature TEV protease and the LysN domain were produced (Fig. 1c). Moreover, the enzymatic activities of the purified TEV proteases released from LysN-TEV and MBP-TEV by autocatalytic cleavage and the commercially available TEV protease (Invitrogen) were compared using LysN-GCSF as a substrate. As shown in Figure S3, the enzymatic activities of three TEV proteases are similar. The results suggest that the soluble TEV protease released from LysN-TEV construct is correctly folded, and that the mechanism of solubility and folding enhancement is similar for different solubility enhancers. In addition, the biological activity of the LysN-GCSF fusion protein was tested on proliferation of target cells. The activity of the fusion protein was about 100 fold lower than the standard possibly due to steric hindrance of the RBD to GCSF receptor binding, but after cleavage with TEV protease the activity increased significantly comparable to that of standard (Fig. 1d). These results suggest that the upstream RBD has the potential to facilitate the proper folding as well as solubility of the downstream proteins in a *cis*-acting manner.

### RNA-mediated protein folding *in vitro*

**Figure 2:**
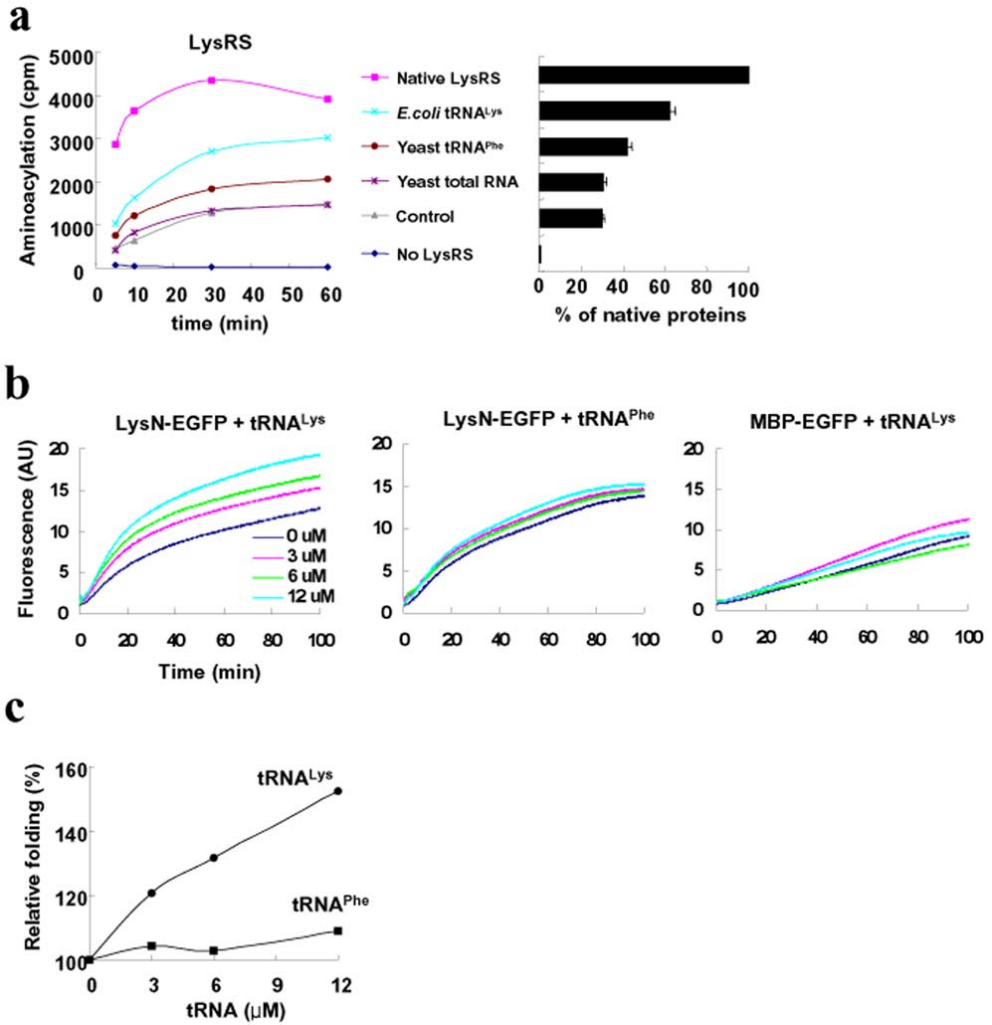
RNA-mediated protein folding *in vitro*. (a) *In vitro* refolding of LysRS. The refolding of LysRS was performed in the presence of *E. coli* tRNA^Lys^ (2 µM), yeast tRNA^Phe^ (2 µM) or yeast total RNA (the amount equivalent to 2 µM of *E. coli* tRNA^Lys^), and then the enzymatic activity of refolded LysRS was investigated using aminoacylation assay, as described in Methods. (b) *In vitro* refolding of LysN-EGFP. Refolding of LysN-EGFP was performed *in vitro* in the presence of *E. coli* tRNA^Lys^ or yeast tRNA^Phe^. The fluorescence emission of refolded LysN-EGFP was continuously monitored. As a control, MBP-EGFP was tested under the same condition. (c) The effects of tRNAs on the refolding yield at 100 min in (b) were compared and summarized. The fluorescence intensity in the absence of tRNA was set to 100%.

To investigate the chaperoning role of RNA to the folding of the RBD-harboring proteins, *in vitro* refolding of LysRS was performed in the presence of cognate tRNA^Lys^ and the activity of refolded LysRS was monitored by aminoacylation assay. The results showed that the folding of LysRS into functionally active form was significantly stimulated by the presence of its cognate tRNA^Lys^ as compared to controls either without RNA or with non-cognate RNAs such as yeast total RNA or yeast tRNA^Phe^ (Fig. 2a). Low, but detectable level of stimulation by yeast tRNA^Phe^ may be due to non-specific interactions among non-cognate tRNA and LysRS, consistent with known nonspecific interactions between non-cognate pairs of tRNA synthetases and tRNAs [32].

Because LysRS is large and dimerized protein, it is rather difficult to directly investigate the role of tRNA in the folding process. To simplify the system, LysN was used as a single independent RBD for further studies. LysN was reported to specifically bind to the anticodon of tRNA^Lys^, with dissociation constant (k_d_) in the range of 10^−4^ M, about 10 fold higher than LysRS [31]. The LysN RBD was fused to enhanced green fluorescent protein (EGFP) for monitoring RNA binding-mediated protein folding. To ensure that the chromophore is not formed, the EGFP fusion protein was initially purified from inclusion bodies and used for the refolding studies. The refolding yield of LysN-EGFP was significantly increased by tRNA^Lys^ in a concentration-dependent manner, whereas the increase of refolding yield by yeast tRNA^Phe^ was only marginal (less than 10%) (Fig. 2b and c). The results suggest that the binding between LysN and its cognate tRNA contribute to the enhancement of refolding of LysN-EGFP *in vitro.* In contrast, the refolding yield of MBP-EGFP was little affected by tRNA^Lys^ (Fig. 2b). These results demonstrate that the binding of tRNA^Lys^ to LysN RBD promotes the folding of downstream EGFP, implying the chaperoning activity of tRNA^Lys^ on the folding of LysN-EGFP.

### RNA-mediated solubility enhancement *in vivo*

**Figure 3:**
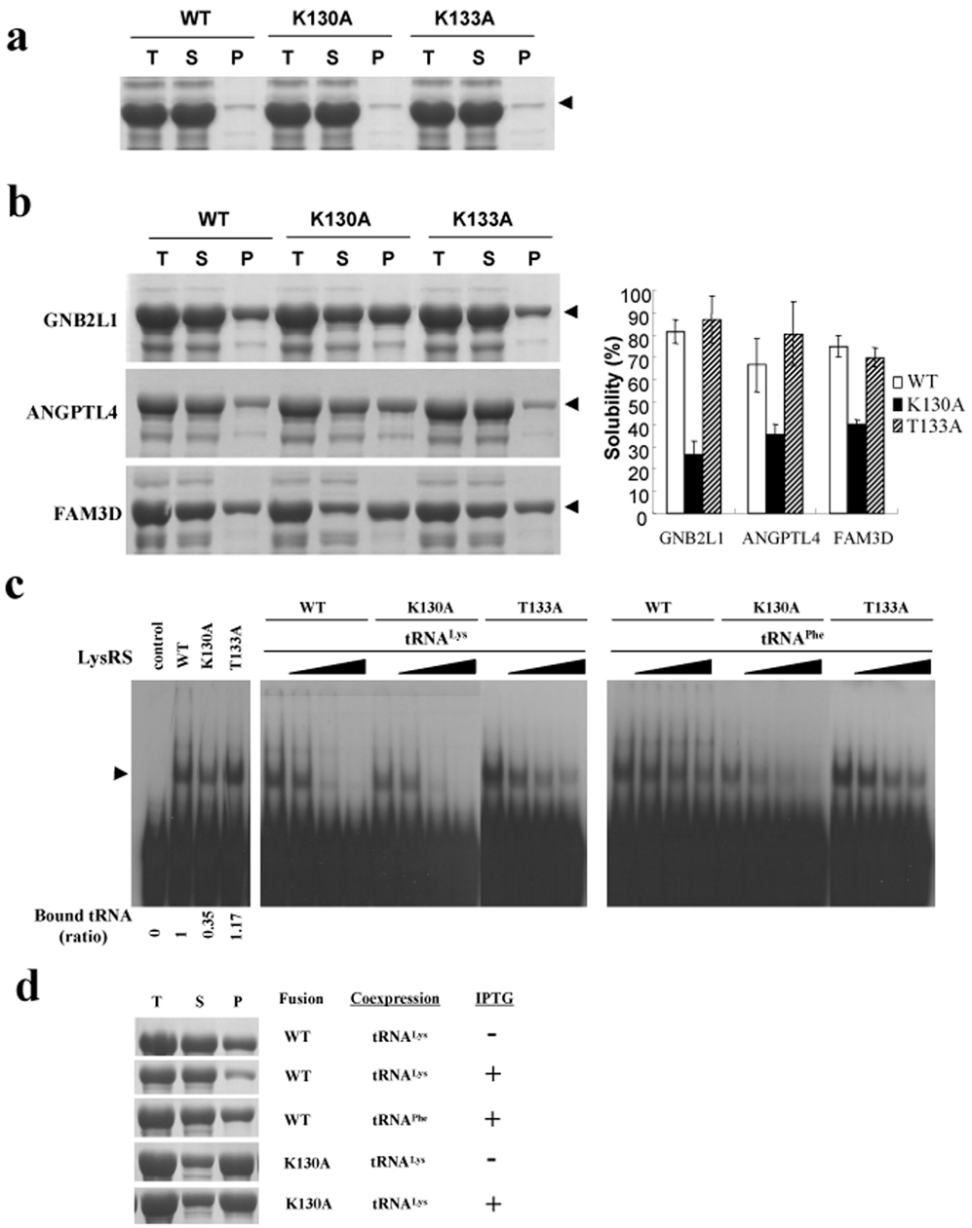
Correlation between RNA binding and solubility enhancement. (a) Expression of unfused wt LysRS, LysRS(K130A), and LysRS(T133A) at 37°C. These proteins have hexa-histidine tag at their C-termini. Mutants were constructed using PCR overlapping mutagenesis. (b) The effects of point mutations on the solubility of LysRS fusion proteins *in vivo*. Three passenger proteins GNB2L1, ANGPTL4 and FAM3D were fused to the C-termini of wt LysRS, LysRS(K130A), and LysRS(T133A). The expression temperature was 37°C (30°C in case of FAM3D fusion proteins). The representative SDS-PAGE data are shown in left panel. The solubility of fusion proteins obtained by three independent experiments is summarized in right panel. (c) RNA binding analysis of LysRS and its mutants using gel-retardation assay. The binding affinity of wt LysRS, LysRS(K130A), and LysRS(T133A) to 5′-^32^P-labeled tRNA^Lys^ was analyzed by gel-retardation assay as described in Methods. For the competition assay, the cold tRNA^Lys^ (middle) and tRNA^Phe^ (right) of various concentrations (0, 0.46, 1.16, and 2.3 µM) was used. Arrow indicates the LysRS-tRNA^Lys^ complexes. Note that the relative amounts of tRNA^Lys^ binding to LysRS, LysRS(K130A), and LysRS(T133A) are 1, 0.35, and 1.17, respectively. (d) The effect of tRNA coexpression on the solubility of LysRS fusion proteins *in vivo*. GNB2L1 as a C-terminal passenger protein was fused to wt LysRS and LysRS(K130A), and the fusion proteins were expressed at 37°C.

Site-directed mutagenesis studies were performed to assess the contribution of tRNA^Lys^ binding to LysRS to the solubility enhancement *in vivo*. The residues at position 130 and 133 in LysRS, predicted to interact with tRNA^Lys^ [31] were replaced with alanine, yielding single point mutants of LysRS(K130A) and LysRS(T133A). The mutations in LysRS at position 130 or 133 in themselves did not affect the solubility of the mutant LysRS proteins (Fig. 3a). We then fused the LysRS mutants with three independent aggregation-prone passenger proteins such as GNB2L1, ANGPTL4 and FAM3D, the information of which are described in detail (Table S1). As shown in Figure 3b, the solubility of LysRS (K130A) fusion proteins was greatly reduced for all three passenger proteins tested whereas that of LysRS(K133A) was not changed or even slightly higher in some cases, as compared with that of LysRS fusion proteins.

We then performed the analysis of the interaction between LysRS and tRNA^Lys^ by gel-retardation assay. The affinity of LysRS(K130A) to tRNA^Lys^ was significantly reduced whereas that of LysRS(K133A) was not or even slightly increased (the amount of radiolabeled tRNA^Lys^ bound to K130A and K133A was approximately 0.35 and 1.17, respectively, when the amounts of bound tRNA^Lys^ to wt LysRS were set to 1 (left panel in Fig. 3c)). The competition assays with unlabeled tRNAs showed that the binding was effectively reduced by the cognate *E. coli* tRNA^Lys^ for all three LysRS constructs, WT, K130A and T133A (middle panel, Fig. 3c) whereas the non-cognate yeast tRNA^Phe^ competed less efficiently (right panels, Fig. 3c). The results in Figure 3 confirm that the solubility enhancement of passenger proteins by LysRS is directly related to the binding affinity of LysRS to tRNA^Lys^.

The contribution of RNA binding to the solubility enhancement was further confirmed *in vivo* by coexpression of tRNA^Lys^. Here, the expression of tRNA^Lys^ was under the control of T7 promoter and induced by IPTG, whereas the expression of LysRS-GNB2L1 fusion protein was under the control of arabinose promoter in a separate vector and induced by arabinose. The coexpression of tRNA^Lys^ significantly increased the solubility of LysRS-GNB2L1 whereas the coexpression of non-cognate *E. coli* tRNA^Phe^ has little or no effect (Fig. 3d). In addition, the coexpression of tRNA^Lys^ did not affect the solubility of LysRS(K130A)-GNB2L1. The results in Figure 3 demonstrate that the binding of cognate tRNA to LysRS plays a key role in solubility enhancement of LysRS-fused passenger proteins *in vivo*.

### Comparison of solubility enhancement between LysRS and MBP

**Figure 4:**
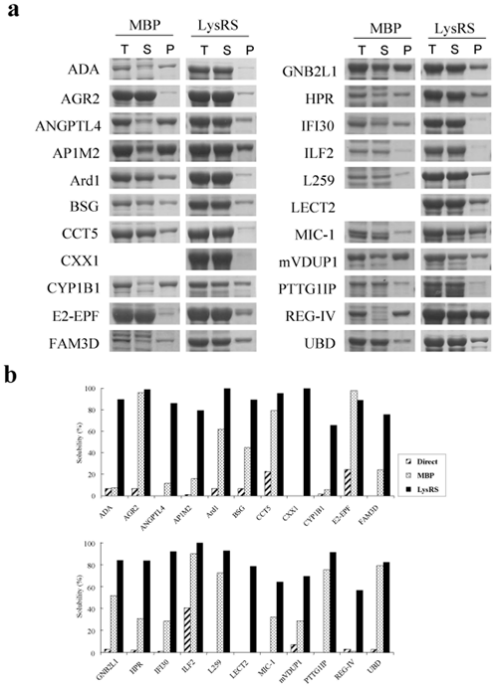
Comparison of the solubility of MBP and LysRS fusion proteins. (a) SDS-PAGE corresponding to the total, soluble and insoluble fractions of fusion proteins. (b) The summary of the solubility of directly expressed proteins and their fusion proteins. The proteins were expressed either at 37°C or at 30°C (AP1M2, CXX1, FAM3D, HPR, mVDUP1). The bands of MBP-CXX1 and MBP-LECT2 were not detectable on the SDS-PAGE. The expression cassette of fusion proteins comprises LysRS (or MBP)-D6-SG-ENLYFQ-MCS-H6 where the sequence of ENLYFQ, MCS, and H6 are TEV protease recognition site, multi-cloning site of *Kpn*I*-BamH*I*-EcoR*V*-Sal*I*-Hind*III, and C-terminal hexahistidine tag, respectively.

If RNA-mediated protein solubility enhancement is what could be observed for RNA binding proteins in general, it could be argued that most of ‘difficult to express’ proteins that by themselves are expressed as misfolded insoluble aggregates could now be expressed as soluble form by fusion to RBD. As a proof of principle, we therefore fused variety of proteins of mammalian origin to RBD and examined the soluble yield and compared with the classic solubility enhancing carrier protein MBP as a control. For this purpose, nineteen human proteins potentially related to the progression of gastric or liver cancers and three mouse proteins were tested [33]–[35]. The information of test proteins is summarized (Table S1). These proteins are diverse in location (cytoplasmic, organellar, and extracellular), pI (lowest pI = 3.94, L259, highest pI = 9.52, MIC-1), and molecular weight (lowest MW = 16 kDa, LECT2, highest MW = 61 kDa, CYP1B1). The test proteins, on direct expression, either failed to be expressed (8 out of 22 cases) or were refractory to soluble expression (14 out of 22 cases) (data not shown). The SDS-PAGE results corresponding to the total, soluble, insoluble fractions of LysRS- or MBP-fused proteins expressed at 30°C or 37°C were shown in Figure 4a. Soluble yields are now compared among the three expression methods (direct expression, LysRS- and MBP-fusion) (Fig. 4b). The results clearly demonstrate that most proteins could be expressed as soluble form by fusion to LysRS, and interestingly enough, LysRS is generally much more superior to MBP for gaining and enhancing the solubility (21 out of 22 cases). It should also be noted that eight of the test proteins (e.g., ANGPTL4, CXX1, FAM3D, HPR, L259, LECT2, MIC-1 and PTTG1IP) failed to be expressed up to detection level when expressed without fusion. This means that the particular RBD used here (*E. coli* LysRS) promotes expression level as well as solubility of passenger proteins.

**Figure 5:**
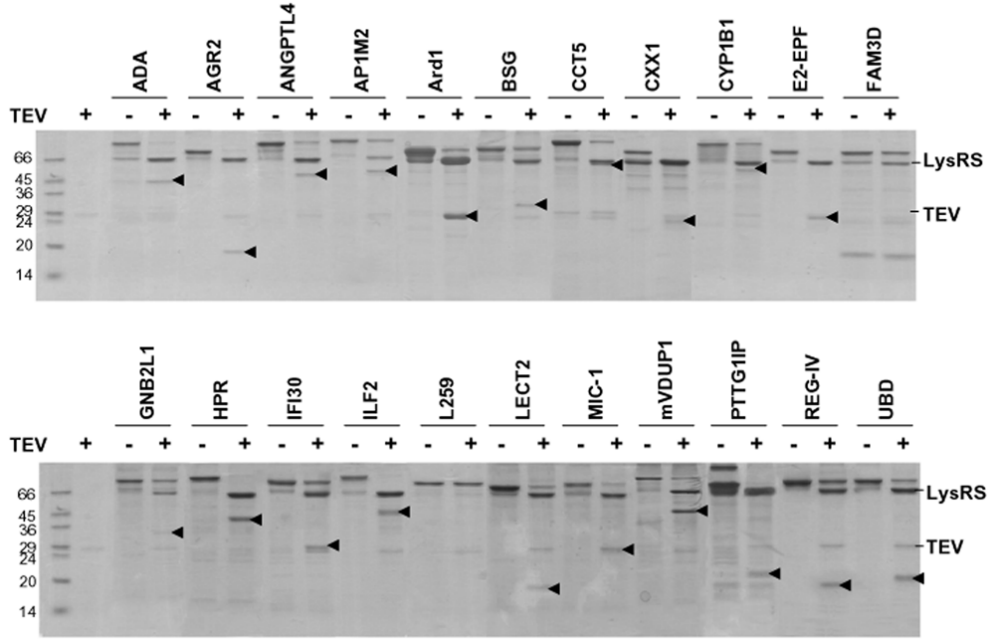
Generation of target proteins from the fusion proteins. Twenty two LysRS fusion proteins purified on one-step Ni-affinity chromatography were incubated at 30°C for 2 h in 30 µl containing 50 mM Tris–HCl, pH 8.0, 0.5 mM EDTA, 1 mM DTT with 1 unit of TEV protease (Invitrogen). All samples were analyzed by SDS-PAGE. The arrow indicates the released target proteins.

All LysRS-fused proteins in Figure 4 were purified via one-step Ni-affinity chromatography. As shown in Figure 5, the target proteins were efficiently released from the fusion proteins by cleavage of TEV protease with minor exceptions (LysRS-FAM3D and LysRS-L259). The results show that the RNA-mediated solubility enhancement is extremely robust for soluble expression of heterologous proteins that are prone to aggregate in *E. coli*.

## Discussion

In this study, we showed that RNA can exert chaperoning effect on the folding of its bound proteins. The result was confirmed through *in vitro* refolding of *E. coli* LysRS and LysN-EGFP in the presence of cognate or non-cognate RNA (Fig. 2) and RNA coexpression *in vivo* on the solubility of LysRS-fused proteins (Fig. 3d). Site-directed mutagenesis of amino acid residues in LysRS involved in RNA binding further confirmed the importance of RNA interaction for the solubility enhancement (Fig. 3b and c). Our results suggest that RNA, a highly soluble polyanionic macromolecule, can increase the solubility of its bound aggregation-prone proteins during the folding process. If the solubility enhancement by RNA is its intrinsic property, the contribution of RNA to *de novo* folding *in vivo* would be greater than we expect, which will be further discussed. Technically, the data presented here further provides a rationale for the development of RBPs as robust solubility enhancers, very useful for high-throughput soluble expression of eukaryotic proteins [36]–[38].

How does RNA promote the solubility and folding of RBD-fused proteins? Polyanionic tags have been known to promote the solubility of their linked proteins [16], [17]. Net charge of solubility enhancers are an important for their solubilizing ability on their passenger proteins [18], [19]. In particular, it was suggested that the electrostatic repulsions of polyanionic surfaces of folded N-terminal solubility enhancer could contribute to the solubility of their downstream polypeptides [18]. It is conceivable, therefore, that the highly negative-charged RNA (75 negative charges in the case of 76 nt long tRNA^Lys^) bound to the folded N-terminal RBD would greatly increase the intermolecular electrostatic repulsions, leading to the promotion of solubility and consequent folding of RBD-fused proteins. This mechanism appears to be in good accordance with the obvious charge effect on protein solubility [12]–[19].

Another possibility to consider is that RNA functions as a specific ligand to bound protein, and the binding of RNA to folding intermediate actually dictates the bound protein to fold into a specific conformation [13], [39], [40]. For example, tRNA^Lys^ (Fig. 2b) might direct folding of LysN of LysN-EGFP, and then the folded LysN might function as a solubility enhancer toward the C-terminal EGFP. It is also possible that folding enhancement of LysN by tRNA^Lys^ prevent unfolded LysN from interfering with folding of down-stream EGFP. However, these explanations does not appear to be satisfactory since LysN and LysRS are known to form their own stable three dimensional structures in the absence of tRNA [30], [31], and LysN alone efficiently fold in a two-state manner *in vitro* [41], although a local ligand-induced (or assisted) folding of LysN and LysRS cannot be completely excluded.

Could the RNA-mediated chaperone-like type be extended to *de novo* folding of native proteins *in vivo*? RNA constitutes a major class of macromolecules inside cells [42], and there are varieties of RNA-binding proteins that generally exhibit significant non-specific affinity [32], which lends credence to ubiquitous nature of RNA-mediated protein folding inside the cells. More importantly, all cytosol-exposed nascent polypeptides on the ribosome of a gigantic RNP complex, prior to formation into stable structure, have been believed to be highly aggregation-prone in the crowded cytosol [43], [44], and are expected to be protected by ribosome-associated molecular chaperones *in vivo* [45]. Extensive analysis so far has revealed that most proteins fold independent of the molecular chaperones [2]–[5], which poses a challenge in *de novo* folding of proteins *in vivo*. So far, however, the potential effects of ribosome, a gigantic RNP complex, on the aggregation behavior of its linked nascent polypeptide have not been given proper attention. The RNP complex (RNA and RBD)-linked aggregation-prone proteins herein described essentially mimics the ribosome-linked nascent polypeptides. Accordingly, it is tempting to speculate that ribosome itself might contribute to the solubility enhancement of its linked aggregation-prone nascent polypeptide in a *cis*-acting manner. If generally large RNA exhibits its intrinsic ability to solubilize its linked polypeptides irrespective of the ligand effect, the present RNA-mediated chaperone type has the potential to play an important role in *de novo* folding inside the cells.

The post-genome research initiatives on structural proteomics require a robust technical platform for protein expression. So far, expression of functionally active proteins in *E. coli* remains a formidable task despite extensive use of molecular chaperones or solubility-enhancing fusion carriers. The soluble expression of variety of proteins of mammalian origin herein presented is extremely robust and could usefully be implemented for high-throughput protein expression for functional and structural genomic research initiatives. While giving new insights into protein folding inside the cells, the present report provides a user-friendly method for protein expression for both analytical level and commercial production and will significant impact on human proteome analysis, target identification and validation for new drug targets.

## Materials and Methods

### Materials

*E. coli* tRNA^Lys^, yeast tRNA^Phe^, and yeast total RNA were purchased from Sigma. The enzymes used for DNA manipulation were purchased from New England Biolabs.

### Construction of protein expression vectors

*E. coli lysS* gene encoding lysyl-tRNA synthetase was cloned into *Nde*I*/Hind*III sites of a derivative plasmid of pGEMEX-1 (Promega) in which one of two *Nde*I sites is deleted, yielding the plasmid, pGE-LysRS. The LysRS expression cassette includes LysRS-enterokinase recognition site-multicloning sites of *Kpn*I*-BamH*I*-EcoR*V*-Sal*I*-Hind*III under the T7 promoter. The plasmid pGE-LysRS was used for the construction of plasmids shown in Figure 1.

Structural genes for *E. coli* C5 of RNase P, Ffh of signal recognition particle, Hsp15, and MBP without signal peptide were obtained by PCR amplification of *E. coli* genomic DNA with the specific primers for each gene. The NP gene of influenza A virus was obtained from PCR amplification of influenza vRNAs using the following primers; 5′ GTC ATC GTC ATC CAT ATG GCG TCT CAA GGC ACC AAA CGC TC 3′ as sense primer, and 5′ GTC ATC GGT ACC ATT GTC GTA CTC CTC TGC ATT GTC TCC 3′ as antisense primer. The obtained PCR fragments encoding fusion partners were cleaved with *Nde*I*/Kpn*I and inserted into *Nde*I*/Kpn*I sites of pGE-LysRS, yielding each fusion vector. The gene encoding tobacco etch virus (TEV) protease with N-terminal histidine tag was amplified using the following primers; 5′ GTC ATCA GGA TCC GGT CAT CAT CAT CAT CAT CAT CAT GGA GAA AGC TTG TTT AAG 3′ as sense primer, and 5′ GTC ATC GTC GAC TTA TTA ATT CAT GAG TTG AGT CGC TTC C 3′ as antisense primer. The TEV protease gene was inserted into *BamH*I*/Sal*I sites of each fusion vector to express TEV fusion proteins. The gene encoding mature GCSF was obtained from the plasmid, pIL20GC [46]. Each gene encoding LysN-EGFP, MBP-EGFP, LysN-TEV, and LysN-GCSF were cloned into the pGE-LysRS.

For the comparison of solubility-enhancing ability between LysRS and MBP in Figure 4, the modified expression vector of pGE-LysRS, which carries LysRS (or MBP)-D6-SG-ENLYFQ-MCS-H6 in place of LysRS-enterokinase recognition site-MCS, was used. However, in case of plasmid pAra-LysRS-GNB2L1 and pAra-LysRS(K130A)-GNB2L1 used in Figure 3d, T7 promoter was replaced with arabinose promoter.

### Construction of tRNA coexpression vector

For coexpression of *E. coli* tRNA^Lys^ or *E. coli* tRNA^Phe^, DNA fragments containing the T7 promoter-matured *E. coli* tRNA^Lys^ (or *E. coli* tRNA^Phe^) gene-T7 terminator was ligated into the *Sal*I*/Sph*I site of plysE (Novagen), yielding pE-tRNA^Lys^ and pE-tRNA^Phe^, respectively.

### Protein expression

The protein expression, SDS-PAGE analysis, and solubility measurement were performed as described previously [18]. Each expression vector was transformed into the *E. coli* expression host, HMS174(DE3)plysE (Novagen). A single colony of transformants was inoculated into 2 ml of LB containing both 50 µg/ml ampicillin and 30 µg/ml chloramphenicol, then diluted into 20 ml of the fresh LB. Cells were cultured till the optical density (OD) reached to 0.5 at 600 nm. Proteins were expressed for 3 h after the addition of 1 mM IPTG. The harvested cells from 10 ml of culture broth were suspended in 0.3 ml of PBS, lysed by sonication. Fifty µl of total lysates was mixed with the same volume of 2 X SDS loading buffer. To separate soluble and pellet fractions, the remaining total lysates were centrifuged at 13,000 rpm for 12 min. The insoluble pellet fractions were resuspended with PBS of the same volume of soluble fractions. Fifty µl of soluble fractions and insoluble pellet fractions were mixed with 50 µl of 2 X SDS loading buffer. After boiling, the samples were loaded and run on SDS-PAGE. The loading amounts of samples were normalized by final cell OD_600 nm_. The gels were stained with Coomassie brilliant blue R-250. The solubility of proteins of interest was estimated on SDS-PAGE using Bio-1D image analysis software (Vilber Lourmat).

To coexpress tRNAs, the RNA expression plasmid (pE-tRNA^Lys^ or pE-tRNA^Phe^) and the protein expression plasmid (pAra-LysRS-GNB2L1 or pAra-LysRS(K130A)-GNB2L1) was co-transformed into the expression host HMS174(DE3). After addition of 0.5 mM IPTG to the growing cells at the OD_600 nm_ of 0.5, the cells were cultured for 30 min, and then 0.02% L-arabinose was added to induce the expression of fusion proteins. After 3h culture, the cells were harvested.

### Purification of proteins

Proteins were purified from 1 L culture of each transformant using nickel affinity chromatography. After addition of 5 ml of the equilibrium buffer A (20 mM Tris-HCl (pH 7.5), 300 mM NaCl, 10% glycerol, 2 mM 2-mercaptoethanol, and 5 mM imidazole) to the harvested cells, the resuspended cells were disrupted by sonication. The soluble fractions were obtained by centrifugation at 30,000 g for 20 min twice and then applied onto HiTrap chelating HP column (5 ml, Amersham Biosciences). After washing, proteins were eluted with 50 ml linear gradients of imidazole ranging from 5 to 300 mM. The fractions containing proteins of interest were pooled and concentrated with Centriprep (Amicon), and dialyzed against the buffer containing 100 mM Tris-HCl (pH 8.0), 100 mM NaCl, 2 mM EDTA, and 2 mM DTT, mixed with the same volume of 100% glycerol. The purified proteins were stored at −20°C until use. For the purification of proteins from inclusion bodies, the cells resuspended in buffer A were lysed by sonication, and then insoluble proteins were obtained by centrifugation. Inclusion bodies were then solubilized in buffer A containing 6 M guanidine-HCl. After centrifugation at 30,000 g for 20 min, the supernatant fractions were collected and loaded on HiTrap chelating HP.

### *In vitro* refolding of LysRS

The purified LysRS with 6 consecutive histidine residue at its C-terminus was denatured in 6 M guanidine-HCl, 1 mM DTT, and 20 mM Tris-HCl (pH 7.8), to a final concentration of 1.3 µM for 2 h at 37°C. The denatured proteins were 50 fold diluted into the refolding buffer containing 20 mM Tris-HCl (pH 7.8), 1 mM DTT, 50 mM NaCl, 1 mM MgCl_2_, and various RNA (2 µM or equivalent to 2 µM *E. coli* tRNA^Lys^) and incubated for 1.5 h at 25°C. The enzyme activity of refolded LysRS was analyzed by aminoacylation assay of LysRS as described previously [25]. The refolding mixture was 10 fold diluted into the aminoacylation assay buffer (total volume of 100 µl) containing 20 mM Tris-HCl (pH 7.8), 150 mM KCl, 2 mM ATP, 0.1 mM EDTA, 7 mM MgCl_2_, 1 µCi of L-[^14^C]-lysine, and 3.7 µM tRNA^Lys^ at 30°C. At different time intervals, 10 µl of reaction mixture was mixed with the same volume of 10% (w/v) ice-cold trichloroacetic acid (Sigma), placed on ice for 10 min. The precipitates were filtered through Whatman No.2 filter paper, and washed once with 95% ethanol, followed by air drying. The bound [^14^C]-lysine was determined with liquid scintillation counter (Beckman).

### *In vitro* refolding of LysN-EGFP and MBP-EGFP

The EGFP fusion proteins purified under the denaturation conditions were incubated in 6 M guanidine-HCl and 1 mM DTT for 20 min at 40°C. The refolding buffer was as described previously [47]. The denatured proteins were 50 fold diluted into the refolding buffer containing 50 mM MOPS (pH 7.0), 100 mM KCl, 5 mM DTT, 5 mM magnesium acetate, 0.2 mg/ml BSA and indicated RNA. The reaction mixtures were incubated at 30°C, and the fluorescence intensity of the refolded EGFP was monitored with excitation at 490 nm and emission at 510 nm using fluorescence spectrophotometer (Varian).

### Cell proliferation assay of GCSF

To investigate the proper folding of downstream protein in RBD-fusion context, *in vitro* assay was performed for both LysN-GCSF and GCSF released from the fusion protein after TEV protease cleavage. LysN and TEV protease were used as control. Mouse myeloid leukemia cell line NFS-60 [48] was cultured in RPMI-1640 containing 10% fetal bovine serum and 2.5 ng/ml GCSF (obtained from CJ Ltd, Korea). After washing the harvested cells with PBS three times, the cells were resuspended in culture medium without GCSF. Test proteins were serially diluted in assay medium, transferred to 96-well plate (50 µl each), and mixed with the same volume of the prewashed cell suspensions in the density of 1×10^5^ cells/ml. Plates were incubated at 37°C for 48 h. After addition of 10 µl of the MTT solution, the plates were further incubated for 4 h before quenching by acidified solution containing isopropanol. The absorbance was measured at 550 nm with ELISA reader (Tecan). The mean value of absorbance was converted to international unit (IU) using the standard GCSF as a reference.

### Gel-retardation assay

*E. coli* tRNA^Lys^ (Sigma) was treated with alkaline phosphatase (Roche). After heat inactivation of alkaline phosphatase, tRNA^Lys^ was labeled with [γ-^32^P]ATP(3000 Ci/mmol) (PerkinElmer) by T4 kinase. The 5′-^32^P-labeled tRNA^Lys^ was purified using Sephadex G25 column (Roche). The interactions of purified LysRS and its variants with radiolabeled tRNA^Lys^ were assayed by gel-retardation assay as described previously [49]. The binding reaction was performed in 20 µl buffer (20 mM Tris-HCl pH 7.5, 150 mM NaCl, 10 mM MgCl_2_, 10 mM 2-mercaptoethanol, 10% glycerol, and bovine serum albumin at 0.1 mg/ml) at 25°C for 20 min. After electrophoresis on 6% polyacrylamide gel containing 5% glycerol and 0.5 X TBE at 4°C, the fixed and dried gels were subjected to autoradiography.

## Supporting Information

Figure S1 Enhancement of solubility of proteins by fusion to RNA-binding proteins. The tested proteins include *E. coli* C5, Ffh of signal recognition particle, and Hsp15. TEV protease was fused to the C-terminus of each RBP. Fusion proteins were expressed at 37°C and 27°C, and the solubility of fusion proteins were analyzed by SDS-PAGE. T, S, and P represent the total extract, soluble fractions, and insoluble fractions, respectively.
(0.62 MB TIF)

Figure S2 Functional assay of RNA-binding protein (RBP)-fused TEV. To check the proper folding of RBP-fused TEV proteins, purified LysN-GCSF fusion protein carrying linker peptide of TEV recognition site was used as substrate. All RBP-fused TEV proteins were purified via nickel affinity column (data not shown). The cleavage reaction was performed in 30 µl of reaction volume containing 50 mM Tris-HCl (pH 8.0), 0.5 mM EDTA, 1 mM DTT, 6 µg of LysN-GCSF as substrate, and each RBP-fused TEV protein for 1h at 30°C. The reaction products were analyzed by SDS-PAGE.
(0.62 MB TIF)

Figure S3 Functional assay of TEV proteases. (a) The tested TEV proteases. The released TEV proteases from LysN-TEV and MBP-TEV by autocatalytic cleavage *in vivo* (nTEV and mTEV, respectively) were purified by one-step Ni-affinity chromatography. The commercially available rTEV (Invitrogen) was used as a positive control. (b) The activities of TEV proteases. The TEV protease cleavage reaction was carried out in 120 µl of reaction volume containing 50 mM Tris-HCl (pH 8.0), 0.5 mM EDTA, 1 mM DTT, 30 µg of LysN-GCSF as substrate, and 2 µg each TEV protease at 30°C. Twenty µl of the reaction mixture was sampled at indicated time intervals (10, 20, 40, and 80 min). These samples and uncleaved substrate (named S) were analyzed by SDS-PAGE. (c) The extent of substrate cleavage was estimated on the above SDS-PAGE by densitometric scanning. In the present experimental conditions, the amount of the cleaved substrate (µg) by one µg of each TEV protease (rTEV, nTEV and mTEV) for 1 min was approximately 0.27, 0.25, and 0.24, respectively.
(1.81 MB TIF)

Table S1 The information of 22 proteins used in Figure 3 and 4. Unigene is a system for partitioning GenBank sequences into a nonredundant set of gene clusters, and Reference sequences (RefSeq) database provides references for transcripts, proteins, and genomic regions on NCBI.
(1.76 MB TIF)
