# Supplementary figures and images for: Protein Solubility and Folding Enhancement by Interaction with RNA
Source: PLoS One. 2008 Jul 16;3(7):e2677. doi: 10.1371/journal.pone.0002677 (PMC2444022; doi:10.1371/journal.pone.0002677)

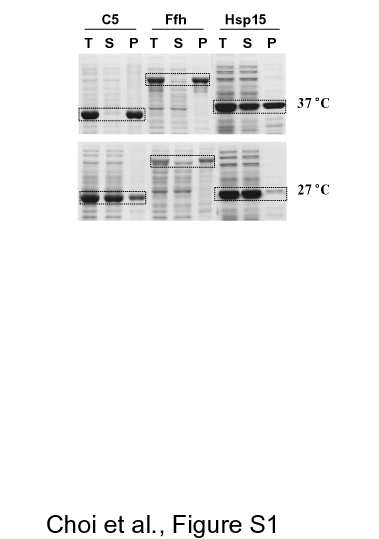

Supplement: Figure S1 — Enhancement of solubility of proteins by fusion to RNA-binding proteins. The tested proteins include E. coli C5, Ffh of signal recognition particle, and Hsp15. TEV protease was fused to the C-terminus of each RBP. Fusion proteins were expressed at 37°C and 27°C, and the solubility of fusion proteins were analyzed by SDS-PAGE. T, S, and P represent the total extract, soluble fractions, and insoluble fractions, respectively. (0.62 MB TIF) [file pone.0002677.s001.tif]

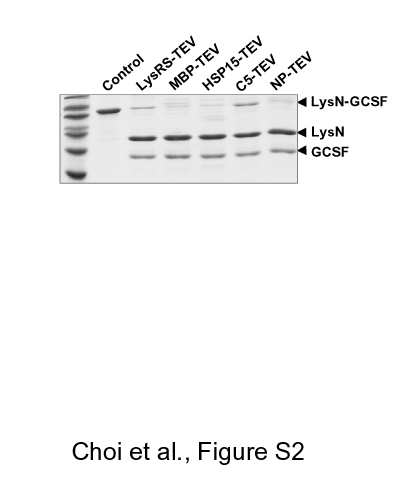

Supplement: Figure S2 — Functional assay of RNA-binding protein (RBP)-fused TEV. To check the proper folding of RBP-fused TEV proteins, purified LysN-GCSF fusion protein carrying linker peptide of TEV recognition site was used as substrate. All RBP-fused TEV proteins were purified via nickel affinity column (data not shown). The cleavage reaction was performed in 30 µl of reaction volume containing 50 mM Tris-HCl (pH 8.0), 0.5 mM EDTA, 1 mM DTT, 6 µg of LysN-GCSF as substrate, and each RBP-fused TEV protein for 1h at 30°C. The reaction products were analyzed by SDS-PAGE. (0.62 MB TIF) [file pone.0002677.s002.tif]

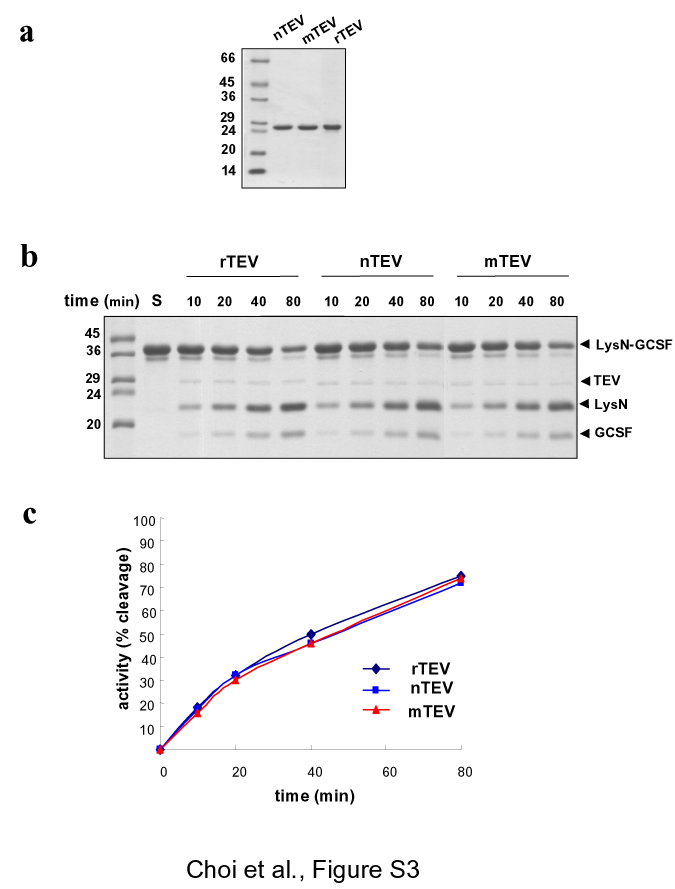

Supplement: Figure S3 — Functional assay of TEV proteases. (a) The tested TEV proteases. The released TEV proteases from LysN-TEV and MBP-TEV by autocatalytic cleavage in vivo (nTEV and mTEV, respectively) were purified by one-step Ni-affinity chromatography. The commercially available rTEV (Invitrogen) was used as a positive control. (b) The activities of TEV proteases. The TEV protease cleavage reaction was carried out in 120 µl of reaction volume containing 50 mM Tris-HCl (pH 8.0), 0.5 mM EDTA, 1 mM DTT, 30 µg of LysN-GCSF as substrate, and 2 µg each TEV protease at 30°C. Twenty µl of the reaction mixture was sampled at indicated time intervals (10, 20, 40, and 80 min). These samples and uncleaved substrate (named S) were analyzed by SDS-PAGE. (c) The extent of substrate cleavage was estimated on the above SDS-PAGE by densitometric scanning. In the present experimental conditions, the amount of the cleaved substrate (µg) by one µg of each TEV protease (rTEV, nTEV and mTEV) for 1 min was approximately 0.27, 0.25, and 0.24, respectively. (1.81 MB TIF) [file pone.0002677.s003.tif]

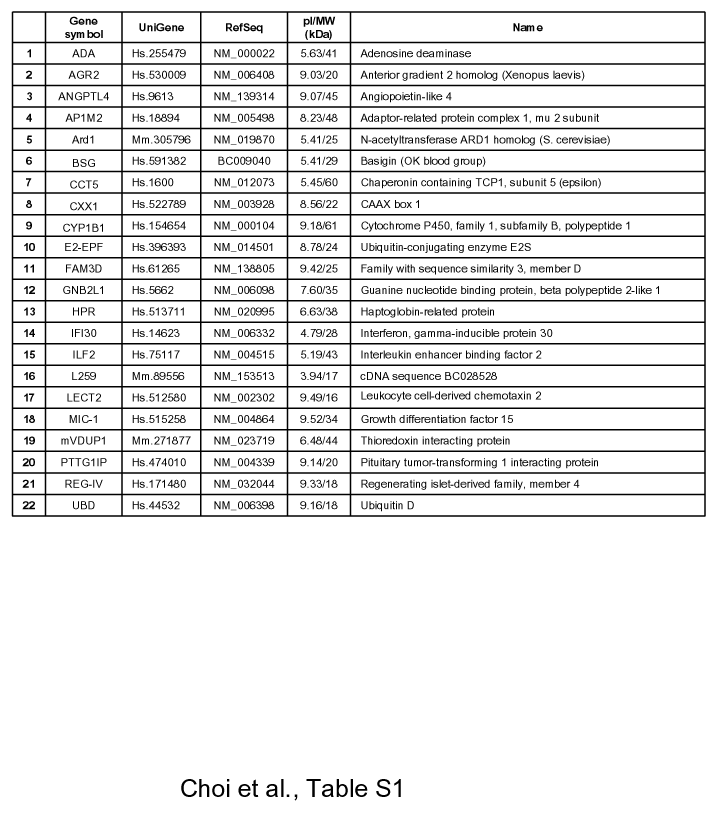

Supplement: Table S1 — The information of 22 proteins used in Figure 3 and 4. Unigene is a system for partitioning GenBank sequences into a nonredundant set of gene clusters, and Reference sequences (RefSeq) database provides references for transcripts, proteins, and genomic regions on NCBI. (1.76 MB TIF) [file pone.0002677.s004.tif]
